# ^124^Iodine: A Longer-Life Positron Emitter Isotope—New Opportunities in Molecular Imaging

**DOI:** 10.1155/2014/672094

**Published:** 2014-05-08

**Authors:** Giuseppe Lucio Cascini, Artor Niccoli Asabella, Antonio Notaristefano, Antonino Restuccia, Cristina Ferrari, Domenico Rubini, Corinna Altini, Giuseppe Rubini

**Affiliations:** ^1^Nuclear Medicine, University of Catanzaro “Magna Graecia”, Viale Europa, Localita Germaneto, 88100 Catanzaro, Italy; ^2^Nuclear Medicine, University of Bari “Aldo Moro”, Piazza Giulio Cesare, No. 11, 70124 Bari, Italy

## Abstract

^124^Iodine (^124^I) with its 4.2 d half-life is particularly attractive for *in vivo* detection and quantification of longer-term biological and physiological processes; the long half-life of ^124^I is especially suited for prolonged time *in vivo* studies of high molecular weight compounds uptake. Numerous small molecules and larger compounds like proteins and antibodies have been successfully labeled with ^124^I. Advances in radionuclide production allow the effective availability of sufficient quantities of ^124^I on small biomedical cyclotrons for molecular imaging purposes. Radioiodination chemistry with ^124^I relies on well-established radioiodine labeling methods, which consists mainly in nucleophilic and electrophilic substitution reactions. The physical characteristics of ^124^I permit taking advantages of the higher PET image quality. The availability of new molecules that may be targeted with ^124^I represents one of the more interesting reasons for the attention in nuclear medicine. We aim to discuss all iodine radioisotopes application focusing on ^124^I, which seems to be the most promising for its half-life, radiation emissions, and stability, allowing several applications in oncological and nononcological fields.

## 1. Introduction

The use of radiopharmaceuticals for molecular imaging of biochemical and physiological processes* in vivo* has evolved into an important diagnostic tool in modern nuclear medicine and medical research. Positron emission tomography (PET) is currently the most advanced molecular imaging methodology, mainly due to its unrivalled high sensitivity, which allows* in vivo* studying of molecular biochemistry. The most frequently used radionuclides for PET have relatively short half-lives (e.g., ^11^C: 20.4 min; ^18^F: 109.8 min) which may limit both the synthesis procedures of different radiopharmaceuticals and the time frame of longer biometabolic processes of* in vivo* studies. Radionuclides of iodine are widely used in nuclear medicine to label monoclonal antibodies, receptors, and other pharmaceuticals in diagnostic and therapeutic applications where quantitative imaging over a period of several days is necessary [1].

Unfortunately, the nuclides most commonly used, ^131^Iodine (^131^I), ^123^Iodine (^123^I), and ^125^Iodine (^125^I), have limitations [2].

Due to its beta emission (606 keV, 90%), ^131^I is often used for therapy. It also emits some gamma photons that can be used for SPECT imaging. Their main emission energy peak is about 364 keV and requires the use of a high energy all purpose (HEAP) collimator. However, its spectrum of emission is complex and some emission peaks, above 364 keV, cause high-energy contamination and degrade dosimetry.

^123^I is more suitable for imaging. The energy of its main gamma emission peak is 159 keV, which is close to the 140 keV from Tc-99m for which gamma-camera design has traditionally been optimized. ^123^I can be imaged in a SPECT system with a low energy high resolution (LEHR) collimator, optimized for the Tc-99m (140 keV), or with a medium energy (ME) collimator, optimized for energies up to 300 keV. However, this radionuclide also emits peaks of a higher energy, which again can be responsible for high-energy contamination [3]. Another limit of ^123^I-labeled radiopharmaceuticals is their short physical half-lives, which permits only the rapid compounds synthesis and the study of short metabolic processes.

^125^I has mainly X-ray energy emission at 27 keV, with low gamma emission at 35.5 keV. It has a photon energy too low for optimal imaging, especially quantitative imaging, and its half-life is undesirably long (42 days). For these reasons, it has not found clinical applications to date [4].

^124^Iodine (^124^I) is an alternative long-lived PET radionuclide attracting increasing interest for long-term clinical and PET studies. ^124^I, a positron-emitting nuclide with a half-life of 4.2 days, could permit PET quantitative imaging over several days. Only about 23% of disintegrations result in positron emission, and these are of relatively high energy [5]. In fact, ^124^I has appropriate physical characteristics, in certain PET radiopharmaceuticals, which can be utilized at low dose; also the spatial resolution of images for this radionuclide is comparable to that obtained with the more conventional PET ones, and the half-life of 4.2 days is appropriate for slow physiological processes also thanks to the clearance of nonspecific radioactivity [6].

PET systems are using an electronic collimation based on interaction time. The emission spectrum of ^124^I is also very complex. In 50% of the cases, the emission of a positron is followed by the emission of a gamma of 602 keV, which is likely to create a false coincidence (similar to scattered coincidences). However, it offers the best image quality due to its electronic collimation, which gives a better efficiency of detection and resolution. The short coincidence window also limits the influence of high-energy peaks. For these reasons, ^124^I seems to be the most promising iodine isotope for an individual pretherapeutic dosimetry [7].

## 2. Physical Data of ^**124**^I

^124^I has dual energy emission: beta radiation emissions of 1532 keV (11%) and 2135 keV (11%) and gamma emissions of 511 keV (46%), 603 keV (61%), and 1691 keV (11%). The gamma constant is 2.05E-4 mSv/hr per MBq @ 1.0 meter. The physical half-time (T1/2) is of 4.18 days, the biological half-time is of 120–138 days, and the effective half-time is of 4 days. The critical organ is the thyroid gland. The radiotoxicity differs if the ^124^I is ingested (2.82E-7 Sv/Bq) or inhaled (1.69E-7 Sv/Bq). The intake routes for ^124^Iodine may be ingestion, inhalation, puncture, wound, and skin contamination [8].

According to these physical properties, ^124^I is the only long-life positron emitter isotope of iodine that may be used for both imaging and therapy as well as for ^131^I dosimetry. The therapeutic effect of ^124^I relies on the Auger electron emission responsible for the local action within a few nanometers; the full cell killing becomes evident when a ^124^I molecule decays within the DNA molecule mainly if placed between the strands.

However, if the ^124^Iodine physical properties are favorable for clinical practice, two major features of the radioactive decay should to be accounted for. One is the image resolution due to the high energy of emitted positron with long range; the other is a high fraction of nonpositron decays accompanied by single photon emission in the same energy window. These factors negatively affect resolution and the signal to noise ratio, as well as the fact that they may limit the quantitative evaluation of PET images. More recently, new algorithms of reconstruction and more specific set-up for isotopes different from ^18^F have been implemented to reduce the image degradation.

## 3. Isotope Production

One of the first schemes for ^124^I production has been based on ^124^Te(d,2n)^124^I nuclear reaction. More recently, however, with the increase in the number of low-energy proton cyclotrons, the ^124^Te(p,n)^124^I reaction has been reaching popularity because it offers the chance of obtaining the highest levels of purity [9]. The two main contaminants affecting iodine preparation are related to the presence of Tellurium isotopes (due to the target physical properties) and to a mixture of ^125^I and ^123^I isotopes for competitive reaction [10]. According to their different half-life, the ^125^I reduces ^124^I purity during time. Because the development of competing reactions may increase with the irradiation energy, it is common to select an optimal irradiation energy window to maximize the production minimizing impurities. Other alternative schemes for ^124^I production include the use of enriched Tellurium (^125^Te (p,2n) ^124^I and ^126^Te(p,3n)^124^I), characterized by higher ^124^I production with a high level of long-life radioactive contaminants, or reactions with nontellurium-based target typically with antimony [11–13]. Factors influencing the ^124^I production are the thermal performance of target, the target composition, and the iodine separation. The target temperature is a critical aspect during production because high yields require high currents of irradiation limited by thermal performance of the target material that may be compensated by an efficient cooling system. Therefore, the irradiation is effective if target temperature is appropriate for thermal performance of target material avoiding volatile isotopes production. Moreover, the thermal stability and performance are related to the chemical form of the target. Commonly, targets for iodine production are composed of Tellurium as TeO_2_ that warrants a good thermal efficiency. More recently, Al_2_O has been added to TeO_2_ providing a better target uniformity, and new promising targets with Al_2_Te_3_ have been proposed [14]. Finally, ^124^I is separated with the dry distillation method by using a vapor pressure in a quartz tube that removes radioactive iodine from the target [15].

## 4. Radiochemistry of ^**124**^I

The iodination procedure with ^124^I has to consider the physical half-life of the radionuclide and the small-scale concentrations and may be performed by using two main types of chemical reactions: electrophilic and nucleophilic substitution (Table 1).

The direct electrophilic substitution approaches were initially developed for protein labeling and later broadened to aromatic compounds. As observed in thyroid hormones synthesis, the tyrosine present in peptides or proteins is the preferred site for radiolabeling. In this chemical reaction, iodide in oxidation state −1 is oxidized by common reagents such as chloramine-T or Iodogen to form a reactive, electrophilic species in the oxidation state +1, which simply substitutes an activated proton from the aromatic ring of tyrosine in the ortho position to the phenol group. The method is simple and usually high yields are obtained. The main disadvantage is the low selectivity if more than one tyrosine moiety is present with also low stability* in vivo*. Moreover, electrophilic radioiodinations can be also performed by means of various demetallation techniques. Demetallation reactions require organometallic compounds as precursors. An important difference from the direct electrophilic substitution is that nonactivated aromatic compounds can be labeled with good or excellent yields. In addition, due to the lack of activating groups, the labeled compounds usually show a much higher metabolic stability when compared to the activated aromatic compounds.

The nucleophilic substitution represents the more simple method for direct iodination using iodide as nucleophile in the oxidation state −1. This exchange reaction can occur in aliphatic and aromatic compounds but proceeds slowly on aromatic ones. An example of this is the exchange of stable iodine, bound to the precursor by radioactive iodine (isotope exchange). This can be achieved by simply heating the components in a suitable solvent such as acetone or water. The consequence, however, is a product of low specific activity and therefore used only in very selected cases, for example, with ^124^I-MIBG.

Finally, other methods have been developed, including the direct labeling of proteins through radioiodination of tyrosine residues with electropositive radioiodine. Chloramine T and various oxidative enzymes are useful oxidizing agents for the in situ oxidation of radioiodine for direct protein labeling. Another approach for iodination employs the prosthetic groups. Prosthetic groups are bifunctional reagents; one allows for high yield radiohalogenation, and the other allows for conjugation to the biomolecule. This strategy became known as the “Bolton-Hunter method” [16].

## 5. Clinical Applications of ^**124**^I

The increased use of imaging with PET is accompanied by a demand for versatile radiopharmaceuticals and positron emitters with relatively long half-life, suitable for the PET procedure and able to highlight the tumor cell characteristics (alteration of enzymology, increased rate of glycolysis, protein and lipid synthesis rate, and DNA syntheses).

Among the radioactive iodine radionuclides using nucleophilic substitution reaction, there are ^124^I-MIBG, ^124^I-IAZA, ^124^I-IAZG, and ^124^I-dRFIB. M-Iodobenzylguanidine (MIBG) is used in diagnosis (labeled with ^123^I or ^124^I) and therapy (labeled with ^131^I) of neuroblastoma and pheochromocytoma [17, 18]. The mean effective dose from ^124^I-MIBG to the adult male human, extrapolated from animal data, was estimated to be 0.25 mSv/MBq. The highest mean equivalent dose was in the thyroid, at 2.343 mSv/MBq [19]. Clinical trials regarding measurements of organ and tumor dosimetry, using ^124^I-MIBG PET/CT in patients with refractory or relapsed neuroblastoma, and the assessment of the accuracy of tumor imaging using ^124^I-MIBG PET/CT versus 123I-MIBG scan with 3-dimensional imaging by SPECT or SPECT/CT by number, intensity of uptake, and localization of sites of tumor are in progress [20]. Moroz et al. described the use of ^124^I-labeled MIBG for imaging norepinephrine transporter (NET) function [21].

^124^I-IAZA and ^124^I-IAZG are hypoxia imaging agents and were used in a comparative study with two other 2-nitroimidazole derivatives (18F-FMISO and 18F-FAZA) for the visualization of tumor hypoxia in A431 bearing mice by means of PET [22].

While ^124^I-dRFIB was synthetized by Stahlschmidt et al. [23] to image cell proliferation, no data on the radiopharmacological evaluation of [^124^I]dRFIB are reported in practice to date.

All the other radioactive iodine radionuclides use electrophilic substitution reaction. One of the first molecules introduced for radiotherapy and labeled with ^124^I was the iododeoxyuridine (IUdR). Radiosynthesis of 5-[^124^I]iodo-2′-deoxyuridine ([^124^I]IUdR) for functional imaging of cell proliferation by means of PET was investigated by Guenther et al. A rapid radioiodination* in vivo*, resulting in high accumulation of activity in the thyroid, was demonstrated. Twenty-four hours after [^124^I]IUdR injection, brain tumor imaging was feasible [24].

Sang et al. described the synthesis of ^123^I- and ^124^I-labeled hypericin derivatives. Hypericin, a natural polycyclic aromatic anthraquinone, was used in the treatment of depression and showed antiretroviral activity against several viruses including human immunodeficiency virus (HIV). Additionally, an elevated activity against protein kinase C (PK-C) was found in malignant gliomas. Sang et al. investigated the possibility of using iodine labeled hypericin derivatives for imaging malignant gliomas with PET and SPECT [25].

Also, ^124^I would be of important value for detecting the expression of successful gene transduction in target tissue or specific organs. Radioiodinated 2′-fluoro-2′-deoxy-1-*β*- D-arabinofuranosyl-5-iodouracil (FIAU) has been used to obtain quantitative* in vivo* PET images of herpes virus thymidine kinase (HSVl-tk) gene expression with superior sensitivity and resolution over that of SPECT ^131^I-radiolabeled FIAU images and also to monitor clinical gene therapy [26, 27].

Studies on central cannabinoid CB1 receptors in schizophrenic patients led to the development of ^124^I-labeled imaging probe n-(morpholin-4-yl)-1-(2,4-dichlorophenyl)-5-(4-[^124^I]iodophenyl)-4-methyl-1hpyrazole-3-carboxamide ([^124^I]AM281). Two ^124^I-labeled cyclin-dependent kinase 4/6 inhibitors were developed to study the role of Cdk 4/6 during cell proliferation in tumor cells. 80% of human tumors show a deregulation of the cell cycle relevant Cdk4-cyclin D1/retinoblastoma (pRb)/E2F signal cascade resulting in uncontrolled tumor growth. Radiolabeled Cdk4 inhibitors have been suggested as promising molecular probes for imaging tumor cell proliferation [28].

^124^I is also important in the study of the expression of multidrug resistance. Colchicine, a potent inhibitor of cellular mitosis, is a member of the multidrug resistance family of drugs. As a potential indicator of resistance, the C-10 methoxy group of n-colchicine has been labeled using 11C- and 13C-iodomethane [29]. However, the restrictions imposed by the short half-life of the carbon-11 compound prompted the investigation into the syntheses of colchicine analogues labeled with radiohalogens.

Iozzo et al. [30] prepared ^124^I-labeled human insulin by direct electrophilic iodination at the A14-tyrosine residue. It retains receptor binding properties and biological activity as the native hormone.

The long physical half-life of ^124^I is particularly well suited for labeling large molecules like antibodies. The relatively long half-life allows PET imaging at late time points (>24 h) ensuring sufficient accumulation of the radiolabeled antibody in the target tissue (e.g., tumor). Various ^124^I-labeled antibodies have been used for molecular imaging and therapy of differentiated thyroid cancer, breast cancer, colorectal cancer, clear-cell renal cell carcinoma, ovarian cancer, and neuroblastoma.

### 5.1. The ^124^I Experience in Thyroid Cancer

^124^I appears as one of the more interesting imaging probes between new agents introduced for PET/CT. It emerged in clinical scenario of thyroid cancer patients because of the high spatial resolution of PET images and better sensitivity than ^131^I [31]. In this setting, ^124^I combines the well-known diagnostic efficacy of iodide family together with the individual dosimetry before ^131^I treatment avoiding cellular stunning.

^124^I in PET is being used mainly in the staging of recurrent or residual thyroid malignancy and for pretherapy individualized dosimetry. Moreover a combined use of ^124^I and ^18^F-FDG PET/CT improves restaging in recurrent differentiated thyroid cancer (DTC). This combination of PET agents is claimed to better predict the outcome of high-dose ^131^I therapy and can be used clinically to decide further management [32]. Moreover in the presence of biochemical recurrence with an increased thyroglobulin (Tg), a negative ^124^I PET can avoid high-dose ^131^I therapy, which implies the need for further additional imaging to estimate iodine nonavid metastatic disease. Phan et al. [33] showed in their study that ^124^I PET detected more abnormalities in comparison to the diagnostic whole body ^131^I scan but showed comparable findings with the posttreatment scan. They reported that only 3 of 11 patients with positive 124I PET scanning, had visible abnormalities in pretherapy scans. Furthermore, PET also proved incremental value by showing lesions in 2 of the 5 patients with undetectable Tg, whereas the whole body iodine scans were negative in all 5 patients. In a comparison between ^124^I and ^18^F-FDG PET in 21 DTC patients at staging or with arising Tg level or with Tg antibodies, without cervical lymph nodes at standard imaging, the reported sensitivities were 80% and 70% for ^124^I and ^18^F-FDG, respectively [32]. The authors reported an incremental diagnostic value for ^124^I when coregistered with diagnostic CT scan. However, near 30% of the lesions were concordantly positive on both PET scans, while positive with only one of these modalities in the others. Authors concluded that the combination of ^124^I and ^18^F-FDG PET/CT improves restaging in recurrent DTC. Although diagnostic role for ^124^I PET should be proven in a large comparable series of patients affected by DTC, the lesion-based dosimetry appears as well defined issue that may be addressed with wide consensus. In fact, by using the lesion-based dosimetry, tumor stunning may be avoided, and replacing the fixed iodine dose, the therapeutic effects are now maximized reducing collateral ones. Maxon using ^124^I has shown that when the radiation dose was greater than 80 Gy, 98% of metastases responded to treatment; on the contrary, only 20% of metastases responded to treatment when the prescribed dose was lower [34]. Furthermore, none of the lesions receiving less than 35 Gy responded to treatment. In a study reported by Dorn et al. in DTC patients over 15 years, they reported in 187 pretherapy ^124^I PET dosimetric evaluations that the delivered ^131^I dose was safe for critical organs (red marrow or lungs) and complete response in metastases was achieved with absorbed doses of >100 Gy [35].

### 5.2. ^124^I-Beta-Cit

**Figure 1:**
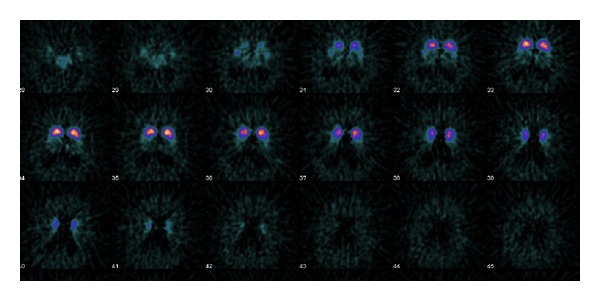
124I-beta-CIT in severe PD. Note a bilateral symmetric involvement of putamen and extrastriatal uptake at 48 h images.

Radiolabeled beta-CIT is often used for brain imaging labeled with ^123^I, but FP-CIT is more suitable for imaging of dopamine transporter because of its higher selectivity and faster kinetics. In particular, beta-CIT is affected in clinical practice by higher affinity for serotoninergic receptors than ^123^I-FP-CIT producing functional images of both neurotransmissions. Moreover, the dopamine binding potential with beta-CIT is obtained after 18 hours from injection, far from an optimal acquisition time for ^123^I-agents. However, beta-CIT has been demonstrated to visualize the dopamine reuptake in different cortical areas, as well as in mesolimbic or mesocortical regions that are not detected by FP-CIT. For these kinetic differences, beta-CIT is considered a very interesting dopamine tracer, affected in practice by physical properties of ^123^I. More recently, we have tested in patients affected by Parkinson's disease (PD) a novel radiopharmaceutical, in which beta-CIT is labeled with ^124^I [36]. It has been obtained by the addition in the following order: 5 mCi of Na^124^I in a solution of 500 *μ*L of NaOH 0.05 N; 50 *μ*g of trialkylstanyl precursor ([2b-carbomethoxy-3b-(4-tributylstannylphenyl)tropane] dissolved, sonicating for 3 minutes, in 150 *μ*L of ethanol; 50 *μ*L of H_3_PO_4_ 0.5 N; 50 *μ*L of CH_3_CO_3_H 0.02 M prepared at the moment of use by 100 *μ*L 32% of peracetic acid dissolved in 2.4 mL of water. After 30 minutes at ambient temperature in an inert atmosphere, we add 100 *μ*L NaHSO_3_ in a solution prepared by dissolving 10 mg in 1 mL. In our unpublished study, we have administered 37 MBq of ^124^I-beta-CIT intravenously in patients affected by PD and essential tremors; then PET images were obtained at 4, 24, and 48 hours from injection. In all patients, we have reported a precise and reproducible evaluation of striatum morphology also in patients with severe PD (Figure 1), as well as of mesolimbic and mesocortical structures probably due to serotoninergic uptake.

## 6. Conclusions

The iodine isotopes with particular regard to ^131^I and ^123^I have represented the cornerstone of nuclear medicine, in thyroid diseases especially. Nowadays, this family has been enriched by ^124^I, the longer positron emitter of iodine, which for the physical properties has gained a role in the clinical practice of molecular imaging.

In fact, ^124^I joins a suitable radioactive emission to a favorable, simple, and well-documented radiochemistry as well as standardized production and target processing. All these aspects together with the high quality of PET technology warrant an actual role in thyroid cancer imaging, as well as promising applications in neurology and oncology.

The future advances in probe development will lead to the production of novel innovative radiopharmaceuticals for specific molecular targeting for both imaging and therapy where the emission of Auger electrons is associated with high resolution quantitative images.

## Figures and Tables

**Table 1:** Principal radiopharmaceutical labeled with ^124^I.

Radiopharmaceutical	Chemical reaction	Function
^ 124^I-MIBG	Nucleophilic exchange	Adrenergic activity
^ 124^I-IAZA	Nucleophilic exchange	Hypoxia agent
^ 124^I-IAZG	Nucleophilic exchange	Hypoxia agent
^ 124^I-dRFIB	Nucleophilic exchange	Cell proliferation
^ 124^I-IUdR	Direct electrophilic substitution on activated aromatic systems	Cell proliferation
^ 124^I-labeled-hypericin	Direct electrophilic substitution on activated aromatic systems	Protein-kinase C
^ 124^I-FIAU	Direct electrophilic substitution on activated aromatic systems	Herpes virus thymidine kinase
m-^124^I-IPPM	Electrophilic radioiododestannylation reactions	Opioid receptors
^ 124^I-IPQA	Electrophilic radioiododestannylation reactions	EGFR kinase activity
^ 124^I-labeled-6-anilino-quinazoline deriv.	Electrophilic radioiododestannylation reactions	EGFR inhibitors
^ 124^I-label.purpurinimide derivatives	Electrophilic radioiododestannylation reactions	Tumor imaging
^ 124^I-label.CDK4/6 inhibitors	Electrophilic radioiododestannylation reactions	Cell proliferation
